# Integrating Clinical Reasoning Into Medical Students’ First Weeks of Education Improves Understanding of Cranial Nerve Anatomy

**DOI:** 10.7759/cureus.70889

**Published:** 2024-10-05

**Authors:** Mario Loomis, Jailenne I Quinones-Rodriguez, Rylie Wackerly, Kathryn B Spears, Teresa Loomis

**Affiliations:** 1 Department of Clinical Anatomy, Sam Houston State University College of Osteopathic Medicine, Conroe, USA

**Keywords:** analytical reasoning, cranial nerve anatomy, foundations of clinical reasoning, interactive educational modules, preclinical medical education

## Abstract

Clinical reasoning is essential to the practice of medicine. Such reasoning involves analytical (deductive) and non-analytical (recall) processes. Non-analytical reasoning is taught extensively in medical schools, and it dominates medical students' time as they review question banks and lecture notes, watch videos online, and memorize flashcards, algorithms, and illness scripts. However, few opportunities are provided in the curriculum to develop students' clinical reasoning skills, and when they are, the diverse levels of innate reasoning ability among students often lead to significant learning disparity. To address this deficiency, a pilot module on cranial nerve anatomy was developed to foster analytical clinical reasoning in an individualized manner. It was hypothesized that this module would not only introduce the foundations of an essential medical skill but also improve overall student understanding of the subject and reduce learning disparities among students. A comparative study was conducted using this module in one group and a didactic module in the other, employing pre- and post-testing measures. Results indicated a 26% improvement in average scores following the analytical module, whereas the control module showed no significant improvement. In addition, the disparity between students improving or not improving following the intervention was reduced, with 74% of students improving after the reasoning module and only 33% of students improving after the didactic module. A novel cranial nerve educational module introduced analytical reasoning in medical students' first few weeks of education, facilitating the learning of complex anatomy and reducing learning disparity between students.

## Introduction

It has been estimated that 25% of medical errors are due to faulty clinical reasoning [1]. Among the various cognitive models of clinical reasoning, script theory and dual process theory emerge as the most favored among medical educators [2]. Script theory proposes that clinical reasoning involves recalling previous patient presentations or illness scripts, which are then "replayed" in the mind, searching for one that fits the current patient. Dual process theory posits two mechanisms of clinical understanding: analytical and non-analytical reasoning. Non-analytical reasoning involves recalling previously learned scenarios, along the lines of illness scripts. On the other hand, analytical reasoning involves a step-by-step deductive process correlating the facets of the patient presentation with foundational concepts. Within these cognitive models, four domains of clinical reasoning have been described: information gathering, hypothesis generation and testing, differential diagnosis generation, and treatment planning [1,3]. The last two domains are beyond the scope of first-year medical students, but the first two, information gathering and hypothesis generation, fall within their capacity. Information gathering is the non-analytical process of recalling previously learned material relevant to a patient presentation. Recall activity resides at the lowest level of Bloom's taxonomy, yet it is how first-year medical students spend most of their time. They memorize and recall names, create flashcards and mnemonics, and run through practice questions. The second clinical reasoning domain, hypothesis generation and testing, resides higher in Bloom's taxonomy in that it involves analytical reasoning. Analytical reasoning can offset the limitations of non-analytical reasoning. Since one cannot see and recall all possible scenarios, reasoning through the evidence is a way to "think outside the box" when a diagnosis does not readily come to mind. It is also a way to analyze and confirm the accuracy of a diagnosis that does come to mind. One of the three most common short-cuts, or heuristics, for recalling an illness script is anchoring, jumping to a previous diagnosis associated with a similar scenario. This is a cognitive bias and as such, it is a potential cause of medical errors [4]. Analytical reasoning approaches the diagnosis from another vantage point, checking for this bias and potential error. Due to the importance of clinical reasoning skills in the practice of medicine, efforts are being made to teach the skill of analytical reasoning in clinical skills courses before students begin their clerkship rotations. We incorporated it even earlier, i.e., in the first weeks of medical school. Our hypothesis was that analytical reasoning was a foundational skill much like knowledge of anatomy, and the two taught together would not only introduce this essential physician skill but would also lead to an improved understanding of the anatomy. We decided to create an analytical module on cranial nerve anatomy since it has historically been a difficult subject for our students to master. In addition, we hypothesized that the module would lead to more equitable learning. Given the diverse levels of innate reasoning ability among students that could lead to learning disparity, the online module was individualized by having gradated levels of complexity leading students through the reasoning process at their own pace.

This research was previously presented as an abstract and poster at the annual scientific meeting of the American Association for Anatomy in Montreal, Canada, on March 25, 2024.

## Materials and methods

Innovative module design 

**Figure 1:**
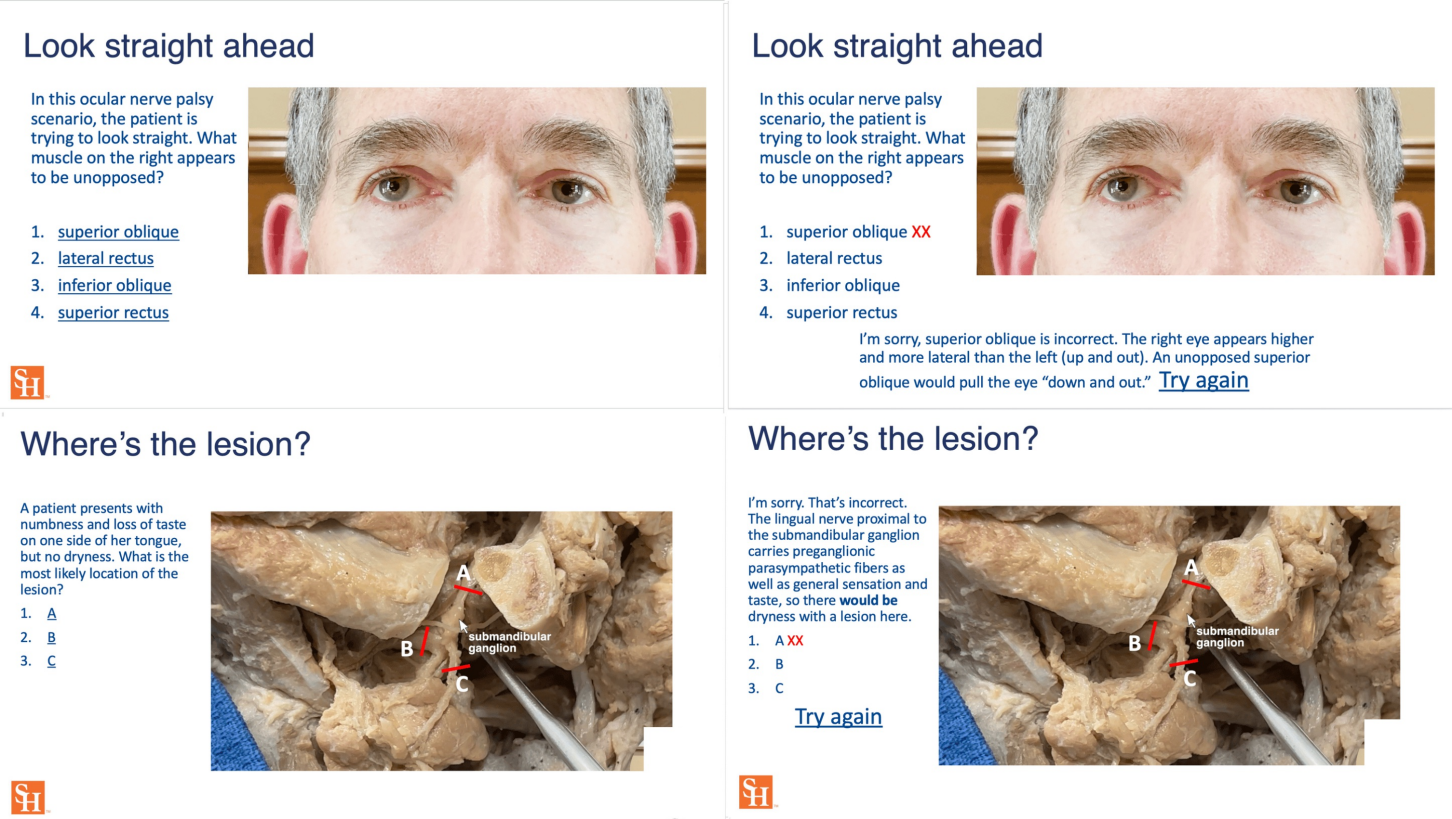
Interactive analytical module. Visual demonstrations with split-screen editing and detailed anatomical dissections guided students through analytical reasoning. The "try again" link led to explanations and additional images and questions when an answer was missed. The figure includes the author’s face to imitate the appearance of ocular palsy.

Both the novel and control cranial nerve modules were deployed prior to the course lectures covering the subject. The novel online module was built with short videos of in situ anatomical dissections beginning with the brain in the cranial vault, following the cranial nerves from their intracranial origins, through the foramina, to parasympathetic ganglia and end organs. The specific areas where autonomic fibers entered and left the cranial nerves were visualized and clinical scenarios were illustrated. Cranial nerves III, IV, and VI were not only visualized in the brain and the skull but their function and dysfunction in various clinical situations were portrayed in videos using split-screen editing to demonstrate various ocular palsies and autonomic imbalances. Interspersed with the videos were interactive slides posing questions that led the students to use the images they had just seen to reason their way to answers, which were then steps along the logical path to diagnosis. When a wrong answer was chosen, it linked to an explanation slide and additional images before going back to the question, facilitating the student's reasoning process and minimizing gaps in their understanding (Figure 1).

This provided a scaffolding of complexity that students advanced through at their own rate and level of understanding, spending more time on one area than another, and covering more basic details when needed. The control module covered the same information, but in a purely didactic format using atlas images, slides, and diagrams. In the control module, students could pause and replay the recording, but there were no questions leading them along the path of logic, and no gradated complexity of material with different pathways of smaller or larger steps in the reasoning process. The control module was essentially a recorded lecture. Both modules had a time limit of 45 minutes to complete. Identical pre- and post-tests were taken by all the students, consisting of five high-order questions with a time limit of two minutes per question. The post-test was taken immediately following the completion of the module (Table 1).

**Table 1:** Pre- and post-module test questions. The same questions were given before and after both the novel and control module. * Correct answer.

Question	Answer choices	
A patient is noted to have anisocoria (unequal pupils) in a dimly lit exam room. When the room lights are brightened, the abnormality corrects itself significantly. Which of the following is the most likely diagnosis?	1. Normal variant. 2. Partial cranial nerve III palsy. 3. Carotid artery disorder*. 4. Ciliary ganglion disorder. 5. Ciliary body disorder. 6. No idea	
Three months after undergoing surgical removal of the superficial lobe of her right parotid gland, a patient notes visible sweating of the right cheek whenever she eats. This phenomenon is due to abnormal innervation of dermal sweat glands by nerves whose cell bodies are in which of the following?	1. Auriculotemporal nerve. 2. Mandibular nerve. 3. Facial nerve. 4. Pterygopalatine ganglion. 5. Otic ganglion*. 6. Submandibular ganglion. 7. No idea	
During the H-test, a patient has the most notable double vision while trying to look to the right and down. If this is due to an isolated nerve disorder, which of the following muscles is most likely not functioning normally?	1. Left lateral rectus. 2. Right medial rectus. 3. Right superior oblique. 4. Left superior oblique*. 5. Right inferior oblique. 6. Left inferior oblique. 7. Cannot tell	
The varicella (chicken pox) virus, having laid dormant in a patient's neuronal cell bodies after an initial infection 30 years earlier, has now become active. It has caused swelling of those neuronal cell bodies with the migration of the virus to the nerves' peripheral endings. The patient has painful blisters on one side of his tongue associated with ipsilateral (same side) facial nerve paralysis. From which of the following sites did the virus most likely originate?	1. Trigeminal motor nucleus. 2. Trigeminal sensory nucleus. 3. Facial motor nucleus. 4. Pterygopalatine ganglion. 5. Geniculate ganglion*. 6. Submandibular ganglion. 7. No idea	
A patient being seen for concerns of diplopia (double vision) enters the exam room holding their head leveled (not tipped up or down) and turned to the left, with eyes looking forward. When asked why she is holding her head this way, she states that it makes the double vision go away. Which of the following is most consistent with this?	1. Left oculomotor palsy. 2. Right oculomotor palsy. 3. Left trochlear palsy. 4. Right trochlear palsy. 5. Left abducens palsy*. 6. Right abducens palsy. 7. No idea	

Participants

All participants (n = 74) were first-year medical students enrolled in the clinical anatomy course. The Sam Houston State University Institutional Review Board approved the study, which classified it as exempt under Category 2 (ii) on May 9, 2023 (Approval Number: 2023-118). This category pertains to research involving only educational tests (cognitive, diagnostic, aptitude, achievement), survey procedures, interview procedures, or observation of public behavior (including visual or auditory). All participants provided informed consent prior to their involvement in the study.

Upon admission, students in the first-year class of 160 were separated into four learning communities (one through four) based only on a balanced gender distribution. Learning communities one and two were combined, as were three and four, to create two experimental groups. One group was offered the novel analytical module and the other, the control module. Participation was voluntary and anonymous, with students completing the modules individually online during their free time. In the novel, analytical reasoning group, 40 students out of 80 agreed to participate. In the control group, 34 out of 80 agreed to participate.

Quantitative analysis

The study employed a two-group, pre-test/post-test design to evaluate the effectiveness of a novel analytical reasoning module compared to a traditional didactic module. Both groups completed a pre-test before engaging with their respective modules and a post-test afterward consisting of the same questions.

All statistical analyses were performed using GraphPad Prism version 10.0 (GraphPad Software, San Diego, CA). A two-tailed paired t-test was conducted to compare the subjective improvement of the learning experience through the novel analytical reasoning and control modules. A repeated-measures t-test using pooled variance compared learning experience improvement within each learning group.

Pre and post-test scores

To assess the efficacy of the novel analytical reasoning module, a two-way ANOVA with Šidák correction was conducted to account for multiple comparisons and control the family-wise error rate. Pairwise comparisons between groups were performed to identify significant differences in pre-test and post-test scores. This approach evaluated the main effects of the type of module (novel vs. control), time (pre-test vs. post-test), and the interaction between these two factors. A p-value of 0.05 or less was defined as statistically significant along with a 95% confidence interval. This threshold was used to test our hypothesis that the novel analytical reasoning module would enhance the overall understanding of cranial nerve anatomy and reduce learning disparities among students.

## Results

**Figure 2:**
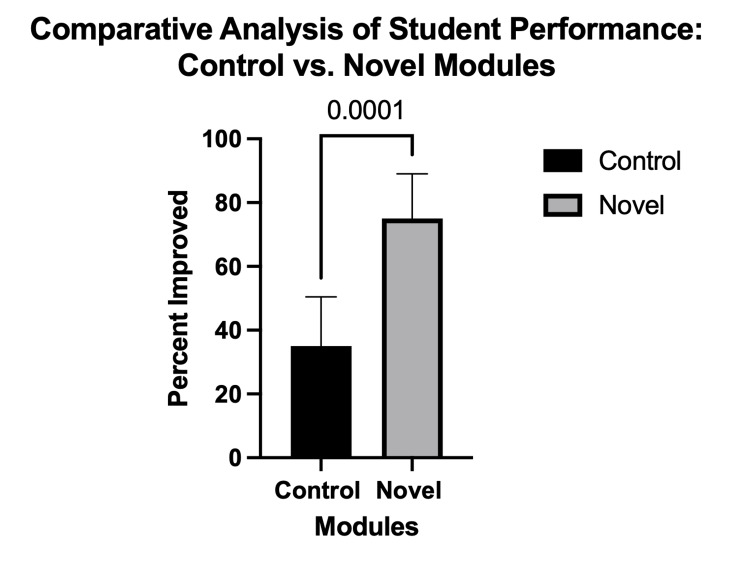
Percentage of students who improved following the module. Following the novel module, 74% of students showed improvement versus 33% following the control. The bar graphs show the quantification. Results are shown as mean ± standard error of the mean (SEM) (n = 74).

The comparison of the control didactic and novel modules in the teaching of cranial nerve anatomy revealed significant improvements across various metrics, demonstrating the efficacy of the innovative approach in enhancing the outcomes for students. The improvement in student scores following the innovative learning module differed significantly from the control group student scores following the didactic module (F (1,40) = 39, CI = (0.2111, 0.5889), p < 0.0001) (Figure 2).

**Figure 3:**
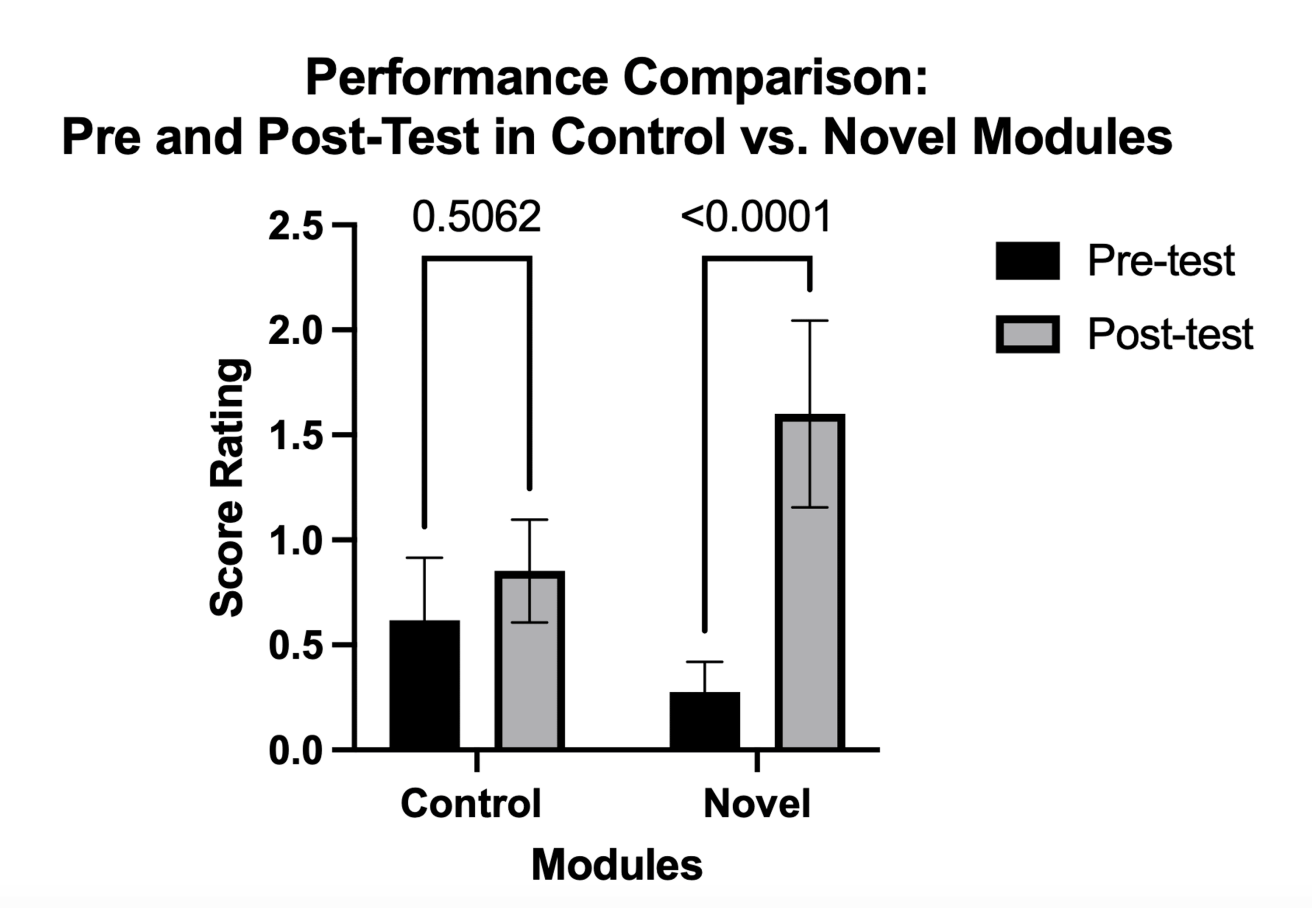
Performance before and after the module. Comparison of the performance of student doctors assessed through pre- and post-test scores, comparing those who participated in control modules against those who engaged with novel instructional modules. The bar graphs show the quantification. Results are shown as mean ± standard error of the mean (SEM) (n = 74).

In examining the performance through between the pre and post-tests, we found an interaction effect between modules. While the average difference between the didactic module pre and post-tests was not significant, the 26 percentage point difference between the reasoning module's pre and post-tests was significant (F (1,34) = 33, p < 0.0001) (Figure 3).

The reasoning module increased interactive engagement, helping the students develop critical thinking and analytical reasoning skills, thereby improving academic performance significantly. This finding supports the hypothesis that modules incorporating analytical reasoning can significantly enhance medical education outcomes. Furthermore, test score improvement among a higher percentage of students following the novel module suggests that its individualized nature may help reduce learning disparities among students, providing a more equitable learning experience.

## Discussion

Anatomy is one of the first courses medical students take in their training, and as such, might be considered elementary when compared to the more complex material covered in systems courses, clinical clerkships, and postgraduate training. However, inadequate anatomical understanding has been directly associated with medical errors, particularly with regard to what has been called "individual anatomy," a patient's particular anatomy that can vary due to congenital, iatrogenic, or pathological influences [5]. It is not enough to simply memorize names and typical relationships. Analytical understanding of anatomy is a central component of the analytical reasoning that underlies sound medical practice [6].

Non-analytical reasoning is primarily what first-year medical students do. They learn to identify and recall the names of hundreds of structures. However, when the functions of structures are taught and clinical correlations are introduced, the potential arises for analytical reasoning within quiz and practice exam problems. Formative questions and practice problems introduce this. Getting a question wrong and searching for the answer has been shown to improve long-term learning [7]. However, as students run through large numbers of practice questions, the process can become more non-analytical, learning the patterns, recalling similar questions, and jumping to a previous answer, rather than reasoning to the answer. Our analytical modules used sequential formative questions as tools to lead students along the stepwise process of reasoning.

At the core of such reasoning skills is the mastery of context [8]. This involves seeing information in light of the big picture or the broad spectrum of potential scenarios narrowed down through reason. This practical reasoning, which one could call medical intuition, is the ability to analyze a presentation and pick out key features to arrive at a diagnosis and plan of action. This underlying concept of context can be integrated into the earliest classes in medical school, particularly those in anatomy. Videos tracing in situ anatomical structures from the brain through cranial foramina, alongside ganglia and adjacent nerves and vessels formed the basis of the novel cranial nerve module in this study. Understanding anatomy in this relational and clinical context is the key to relating structure and function, as well as cause and effect in clinical correlations.

Without context, when fine anatomical details are learned, whether by meticulous cadaver dissection, three-dimensional models, or virtual reality, students learn structures in isolation. The heart and lungs are examined on a tray and virtual images of them are viewed from every angle, but the interplay between the heart, lung, thorax, and adjacent neurovascular structures is lost. The concept of how a mediastinal shift reduces venous return to the heart remains as abstract as before the session. When analytical reasoning is integrated into foundational anatomy, the structures are considered in context. The sympathetic and parasympathetic pathways to the eye are considered in the face of unequal pupil sizes, deducing which pupil is abnormal, whether the sympathetic or parasympathetic tone is diminished, and where along the anatomical pathways the pathology might be.

Clinical reasoning skills are expected of graduating medical students, who gain experience with this skill during their clerkship years using virtual and in-person patient experiences [9,10]. This mental skill, like anatomical knowledge, is a foundational tool used throughout a physician's career. In a recent study that proposed a guide to educational priorities based on a database of malpractice claims, diagnostic errors regarding strokes and eye disorders were among the top 15 most prevalent malpractice claims [11]. These failures to recognize which cranial nerve is dysfunctional and where involve deficiencies in the same type of analytical reasoning as was introduced into this study's novel module.

Many studies have examined different approaches to teaching analytical reasoning, with most programs being carried out during the clerkship years and during graduate medical education [1,12,13]. Some have begun to teach reasoning skills in the preclinical years, specifically in the pre-clerkship curriculum, with signs of success [14]. Such reasoning skills have been taught to second-year medical students with demonstrable improvement in diagnostic skills assessment [15]. However, programs introducing analytical reasoning into the early weeks of anatomy education have not been previously described.

In the current era of artificial intelligence (AI), it might be questioned whether human deductive reasoning is still needed. AI rapidly pulls together related diagnoses to consider, and it has outperformed physicians in picking up lesions on screening medical imaging examinations [16]. At the same time, however, AI has also come up with impressive-sounding but completely false statements, called "hallucinations" [17]. Much like the human error of anchoring, computer algorithms can erroneously correlate online events or statements based on similar appearances. AI, as a powerful form of non-analytical reasoning, must be verified with analytical reasoning [18]. As AI becomes more widely utilized, the need to teach analytical reasoning becomes even more critical.

Our use of anatomical dissection videos in the reasoning module has significant support in the literature, despite the trend to reduce gross anatomy's place in medical school curricula [19]. Supplemental learning technology, such as 3D models, interactive 3D software, virtual anatomy, and mixed-reality programs, while successful at helping students understand three-dimensional structures, have their limitations [20-23]. They are all illustrations studied in isolation. Constructed from medical drawings, not actual anatomical structures, they lack a certain reality and, more importantly, the context essential to analytical reasoning. Overlying and adjacent structures are "deleted," and anatomical context is removed to see deeper structures and systems. Cadaver dissection is still considered the gold standard for learning anatomy, especially for focused individualized review prior to surgery or other medical intervention [24], and prosections and videos of prosections, such as those used in our module, have been found to be as effective as dissection at teaching anatomy [25-28].

Beyond teaching the names and visual appearance of anatomical structures, the novel module was designed to use those structures and their interrelationships to foster analytical reasoning. To do this, the modules were made interactive. Audience response systems can be seen as the modern-day equivalent of the Socratic dialogue, the classic teaching of analytical reasoning. In fact, the use of audience response systems in lectures has increased engagement and measurable learning performance [29]. Such polling questions have also been integrated in a gradated manner called "scaffolding" to foster students' development of analytical reasoning. Improved outcomes on board exams were noted after questions were added to lectures with scaffolding, beginning with foundational concepts and transitioning to higher-order questions [30]. However, there is a disparity in the benefits of in-class interactivity. When interactive reasoning is added to lectures, the more advanced students become bored if steps in the scaffolded reasoning process are made too slowly. If too quickly, then a large class segment gets left behind, losing the chance to reason their way to the answer. Instead, they simply see it displayed on the screen or hear it called out, leaving them only with non-analytical reasoning, another practice question to memorize, another recalled answer that was arrived at by others. While the average performance on board exams may be improved, there are still some who are left behind.

To address this disparate development of analytical reasoning skills among students, the novel cranial nerve module led students through scaffolded steps of reasoning in an individualized manner. Each student did the online module on their own. Different reasoning threads taking smaller or larger steps in the reasoning process were linked throughout the module such that each student advanced at their own pace based on their answering correctly or incorrectly. This minimized gaps in understanding and helped more students arrive at the correct answer through their own analytical reasoning, learning not just the right answer, but the logic behind the answer.

Limitations of this pilot program include the short-term nature of the pre- and post-testing, the relatively small sample sizes, and the lack of long-term follow-up. Future work should assess long-term retention of the benefits and the effects on students' analytical reasoning skills in clerkship years, as well as in second and third-level licensing examinations.

## Conclusions

Learning cranial anatomy is challenging for students due to its complexity and the detailed knowledge required for clinical practice. Despite these difficulties, a thorough understanding of cranial nerve anatomy is essential for conducting accurate physical examinations and for the effective practice of medicine. The introduction of an innovative analytical reasoning module in the first weeks of anatomy has demonstrated substantial improvements in student understanding of complex cranial nerve anatomy with an increased ability to analyze and diagnose complex patient presentations. It also reduced learning disparity among students, with a higher percentage of the participants experiencing improved outcomes.
